# FACILITATORS OF DEPENDENCY IN THE VERY OLD: RESULTS FROM THE NEWCASTLE 85+ STUDY

**DOI:** 10.1093/geroni/igz038.906

**Published:** 2019-11-08

**Authors:** A Kingston, Louise A Robinson, Rachel Duncan, Carol Jagger

**Affiliations:** 1 Newcastle University, Newcastle upon Tyne, United Kingdom; 2 University of Newcastle, Newcastle upon Tyne, England, United Kingdom; 3 Newcastle University, Newcastle upon tyne, England, United Kingdom

## Abstract

In order for governments to plan health and social care strategies to help people maintain independence, evidence is required to show how risk factors are associated with progression in dependency. We use a transparent measure of dependency, based on help needed with activities of daily living, incontinence and cognitive impairment, categorised as: high (24-hour care); medium (daily care); low (less than daily) and independent, then characterise changes over ten years (age 85-95) using the Newcastle 85+ Study while exploring how eight disease groups, multimorbidity and impairments interact to increase care needs. Stroke and diabetes confer an increased risk of low-level dependency. Complex multimorbidity, or three or more falls engendered the greatest risk of transitions to substantial dependency. There should be a focus on prevention of, and appropriate and efficient service provision for those with complex multimorbidity with emphasis on stroke, diabetes and falls, to maintain the independence of older people

